# MQD—Multiplex-Quadrature Detection in Multi-Dimensional NMR

**DOI:** 10.1002/cphc.201100525

**Published:** 2011-11-16

**Authors:** Judith Schlagnitweit, Michaela Horničáková, Gerhard Zuckerstätter, Norbert Müller, Dedicated to Philip W. Kuchel on the occasion of his academic anniversary.

**Affiliations:** [a] Institute of Organic Chemistry, Johannes Kepler University LinzAltenberger Str. 69, 4040 Linz (Austria), Fax: (+43) 732 2468 8747 E-mail: norbert.mueller@jku.at; [b] Kompetenzzentrum Holz GmbH, Science ParkBauteil 2, 2. Stock, Altenberger Straße 69, 4040 Linz (Austria)

**Keywords:** accelerated acquisition, axial peak suppression, multi-dimensional NMR, multiplex-phase cycling, NMR spectroscopy

## Abstract

With multiplex-quadrature detection (MQD) the tasks of coherence selection and quadrature separation in *N*-dimensional heteronuclear NMR experiments are merged. Thus the number of acquisitions required to achieve a desired resolution in the indirect dimensions is significantly reduced. The minimum number of transients per indirect data point, which have to be combined to give pure-phase spectra, is thus decreased by a factor (3/4)^*N*−1^. This reduction is achieved without adjustable parameters. We demonstrate the advantage by MQD 3D HNCO and HCCH-TOCSY spectra affording the same resolution and the same per-scan sensitivity as standard phase-cycled ones, but obtained in only 56 % of the usual time and by resolution improvements achieved in the same amount of time.

## 1. Introduction

### 1.1. Accelerating Multi-Dimensional NMR

Multi-dimensional NMR spectroscopy is an essential tool in chemistry and structural biology. Recent efforts to improve on the methodology target its main weaknesses: low sensitivity and long time requirements. Continuous innovations in NMR hardware (e.g. higher magnetic field strengths, cryogenically cooled probe circuits and preamplifiers,^1^ and the upcoming very promising hyper-polarization techniques^2^, ^3^) have alleviated the basic sensitivity bottleneck for many NMR applications. Given sufficient physical sensitivity, the time required for a multi-dimensional NMR experiment is mostly determined by two factors: 1) the number of indirect time increments, which grows exponentially with the number of dimensions and 2) the coherence selection process. Presently, there are a number of different approaches to reduce total experiment times by acquiring the same information in less time. Prominent examples are sparse-sampling techniques,^4^ single-scan multi-dimensional NMR,^5^ and relaxation optimization as, for example, in fast heteronuclear single quantum coherence (FHSQC) schemes.^6^

Our goal is improving the *overall* acquisition efficiency by reducing the total number of acquisitions required. At the same time information is retained, which is often discarded prematurely in today’s routine pulse sequences, either by gradient defocusing during evolution, or by linear combination during acquisition. We strive to increase the coherence selection efficiency in phase cycling^7^ based on concepts by Ivchenko et al.^8^ They obtained pure-phase 2D spectra of solids by different linear combinations of phase-shifted N- and P-type spectra derived from a single raw data set. Previously we used cogwheel multiplex-phase cycles in homonuclear 2D correlation spectra.^9^ Multiplex-quadrature detection (MQD) is introduced herein as a way to accelerate common multi-dimensional NMR experiments, in particular heteronuclear correlation experiments, utilizing the nested multiplex-phase cycling.

### 1.2. Quadrature Detection in the Indirect Dimensions

Quadrature detection in the indirect dimensions is an important prerequisite to obtain pure-phase spectra. It can usually be achieved either by the States method,^10^ that is, acquiring a pair of free induction decays (FID) with and without a relative 90° phase shift between the excitation and mixing blocks, or by acquiring separate echo and anti-echo FIDs (N/P-selection),^11^ with subsequent linear combination. Many variants of these schemes have been developed over time,^11^, ^12^ like time proportional phase incrementation (TPPI),^13^ States-TPPI,^14^ and echo/anti-echo (EA)-TPPI. Essentially they can all be traced back to one of the two basic schemes. The minimum total number of 1D FIDs, *n*_tot_, required to obtain a phase-sensitive *N*-dimensional NMR spectrum, is thus determined by the length of the basic phase cycle *M* and the numbers of points *n_i_* required to achieve the desired resolution in each indirect dimension as shown in Equation (1):


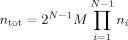
(1)

The factor 2^*N*−1^ is due to the quadrature detection requiring acquisition of one pair of FIDs (either sine and cosine modulated or echo and anti-echo) for each time increment in each indirect dimension.

Echo/anti-echo based schemes can be implemented easily by applying pulsed field gradients during evolution^15^ requiring only two FIDs per time increment but at the cost of irreversibly defocusing the complimentary component in each transient, that is, discarding 50 % of useful signal, thus losing ca. 30 % of the per scan signal-to-noise ratio. In the quest for ultimate sensitivity and efficiency we focus on generally applicable approaches and therefore exclude methods with gradients during evolution times. It is noteworthy, that in conventional implementations of phase-cycled experiments, such waste of potentially useful information is also common, since the FIDs from individual steps are often co-added immediately. Sensitivity improvement techniques as introduced by Kay et al.^16^ alleviate the sensitivity loss in experiments with gradients during evolution for some multi-dimensional heteronuclear experiments. But since the same modifications can also be applied to the corresponding phase-cycled correlation experiments,^17^ the MQD gain in per-scan sensitivity, as outlined below, can also be applied to these sensitivity improved experiments.

Typically multi-dimensional heteronuclear chemical-shift correlation experiments in NMR, like the ones most frequently used for bio-macromolecules, consist of an excitation block followed by a series of single-quantum coherence evolution periods connected by mixing sequences tailored to the specific information sought. Herein we focus on the common type of experiment, where multiple single-quantum evolution times occur. Apart from the specific requirements of the mixing blocks, the basic conventional phase cycle derives from a two-step phase alternation for each indirect dimension to suppress axial peaks, which adversely affect spectral quality even when pushed to the edges of the respective indirect dimensions by use of TPPI.^13^ A 180° phase change of the pulse or block preceding an evolution period is applied in concert with receiver phase inversion in order to suppress the pathway through coherence order zero, which causes axial peaks in the respective dimension, such as seen in cases of non-perfect 90° pulses or fast relaxation, for example, in paramagnetic proteins.^18^ As outlined above, to achieve quadrature separation usually, for each increment of each indirect dimension, two FIDs with 90° shifted relative phases of the coherence-transfer pulses are acquired separately. In the original TPPI method^13^ this doubling of recorded transients is masked by a virtual doubling of the spectral width, which however has the same overall effect of doubling the experiment duration. Thus, excluding gradient selection during evolution periods for the reasons given above, the minimum total number of acquired transients in an *N*-dimensional NMR experiment scales with 4^*N*−1^ (that is 2^*N*−1^ for the basic axial peak suppression phase cycle, which is included in *M* in Equation (1), times 2^*N*−1^, for the quadrature detection). The decrease of *n*_tot_ described in Section 2 corresponds to reducing the factor 2^*N*−1^×*M* by merging coherence selection and quadrature separation in a multiplex manner.

## 2. Results and Discussion

### 2.1. Multiplex-phase cycling to Achieve Quadrature Separation

**Figure 1:**
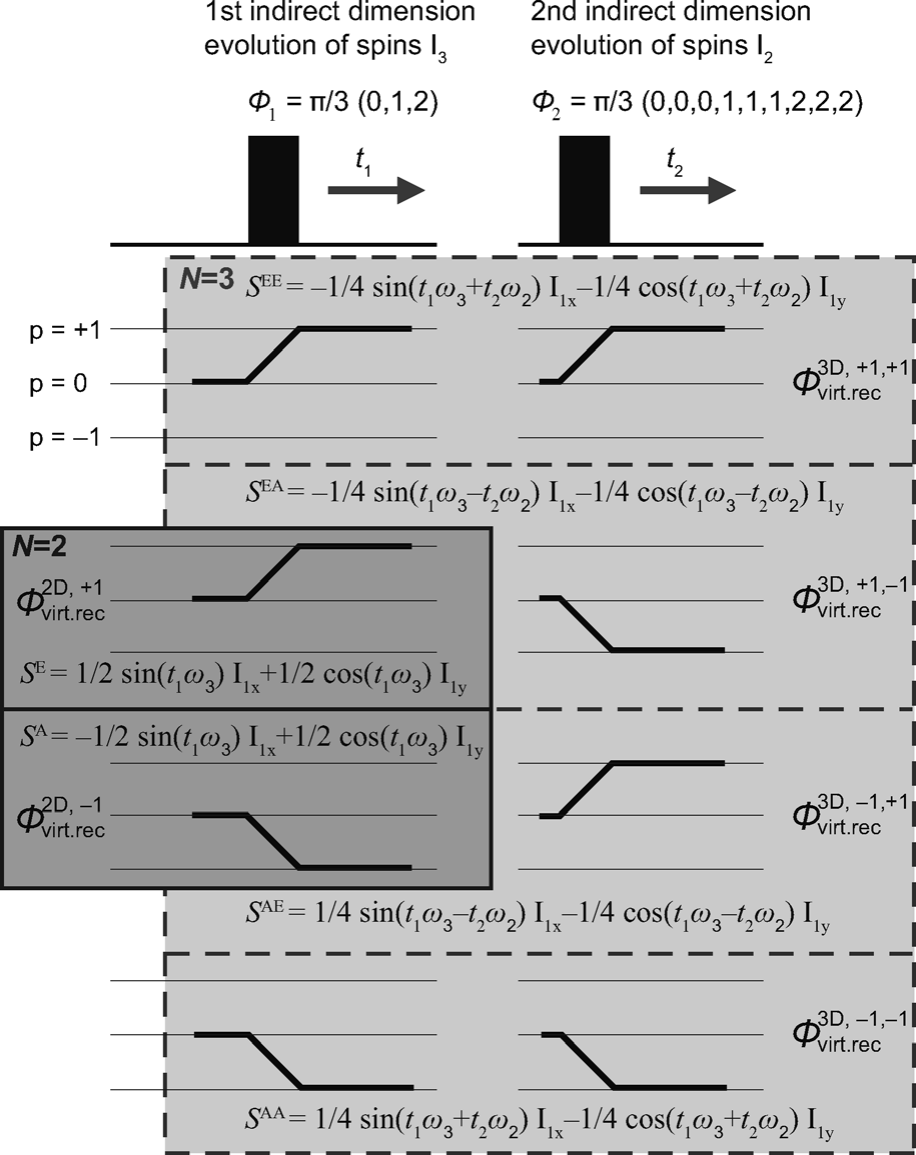
Graphs of the indirect evolution parts of an *N*-dimensional pulse sequence. Cartesian product operators (computed with POMA^24^) and coherence pathways (from CCCP^23^) are shown for one and for two indirect dimensions. In case of *N*=2 a three-step phase cycle *Φ*_1_ on one 90° pulse is sufficient. As shown by the product operators and the coherence-transfer pathways in the dark grey box, using different receiver phase cycles *Φ*^2,+1^=(2π/3) (0,2,1) and *Φ*^2,−1^=(2π/3) (0,1,2) either the echo or the anti-echo pathway can be selected. In case of a 3D experiment the phase cycle is extended to nine steps since a second pulse has to be phase cycled (*Φ*_2_). All possible combinations of echo and anti-echo pathways in the two indirect dimensions can be selected with the receiver phase cycles *Φ*^3,+1,+1^=(2π/3) (0,2,1,2,1,0,1,0,2), *Φ*^3,+1,−1^=(2π/3) (0,2,1,1,0,2,2,1,0), *Φ*^3,1,+1^=(2π/3) (0,1,2,2,0,1,1,2,0), and *Φ*^3,−1,−1^=(2π/3) (0,1,2,1,2,0,2,0,1).

Cycling the phase(s) of the excitation pulse(s) preceding the indirect dimension in a 2D NMR experiment in three (or more) steps allows one to select either coherence order *p*=1 or *p*=−1 by changing only the receiver phase (Figure 1). Following the fundamental idea of multiplex-phase cycling^8^ one can store the FIDs corresponding to each phase-cycle step separately in a raw data set (advantageously in an interleaved manner), while the spectrometer receiver phase is unchanged. The receiver phase shift can then be achieved by post-acquisition computation. Coherence selection is consequentially accomplished by complex linear combinations of these primary raw FIDs. Different linear combinations, corresponding to different selections of coherence-transfer pathways, can thus be obtained without additional acquisitions. For this purpose the signal *S*_m_ obtained for each phase-cycle step *m* is multiplied by a complex coefficient 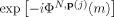. These coefficients depend on the *virtual receiver phase cycle* 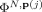
for an *N*-dimensional experiment selecting coherence order **p**(*j*) during the indirect evolution period *j* (*j* taking values from 1 to *N*−1). The post-acquisition phase-shifted FIDs of the *M*-step phase cycle are then combined, according to the Equation (2):^8^


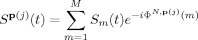
(2)

Cycling the excitation-pulse phase in three steps of 120° for each indirect dimension gives an overall number of 3^*N*−1^ FIDs, which are recorded and stored separately and combined to the echo [**p**(1)=+1] and anti-echo [**p**(1)=−1] linear combinations for each time increment. Alternatively a cogwheel phase-cycling scheme^19^ can be used, which may be beneficial for multiple-quantum evolution times.

The echo FID *S*^+1^ and the anti-echo FID *S*^−1^ are calculated as shown in Equations (3) and (4):


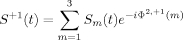
(3)


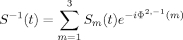
(4)

using two virtual receiver phase cycles with *Φ*^2,**+1**^=(2π/3) (0,2,1) and *Φ*^2,**−1**^=(2π/3) (0,1,2) on the same three *actual* FIDs. After the linear combination the echo and anti-echo FIDs for each time increment of the indirect dimension are stored alternately. This procedure yields an echo/anti-echo data set, which can be processed in the conventional way and leads to pure-phase spectra. In Figure 1 the excitation-pulse phase cycle for recording the raw data set and the coefficients representing the virtual receiver phase cycles, which are used to calculate the linear combinations to give the echo and anti-echo parts, are shown for 2D and 3D experiments.

This concept can be expanded straightforwardly to higher- (*N*-) dimensional experiments by constructing nested three-step phase cycles for each indirect dimension. With nested three-step multiplex-phase cycling, the minimum total number of FIDs is 3^*N*−1^, giving a time reduction by a factor (3/4)^*N*−1^ as compared to the conventional phase cycled States or EA experiment. A number of scans smaller than this is only possible, when compromising either the per-scan sensitivity (e.g. by gradients during evolution) or coherence selectivity (e.g. by omitting axial peak suppression). The benefit is bigger for higher-dimensional experiments, since spectra with the same sensitivity and resolution can be obtained in significantly less time (i.e. for 2D, 3D, 4D, 5D and 6D spectra only ca. 75 %, 56 %, 42 %, 32 %, and 24 % of the respective time is needed). Basically these time savings are achieved by combining coherence selection and quadrature separation. Keeping the total experiment time constant, this saving can of course be utilized to increase the resolution in the indirect dimensions. Moreover, this approach can probably be combined with other acquisition accelerating techniques, such as non-uniform sampling,4d band-selective optimized-flip-angle short-transient (SOFAST),^20^ reduced dimensionality,4a projection reconstruction4b as well as transverse relaxation optimized spectroscopy (TROSY),^21^ and cross relaxation-enhanced polarization transfer (CRINEPT).^22^

To assess the selection efficiency of the multiplex-phase cycled experiments the programs CCCP^23^ (for simulating coherence-transfer pathways) and POMA^24^ (for Cartesian product operator calculations^25^) were used. The results of the MQD pulse sequences for basic 2D and 3D NMR experiments are summarized in Figure 1. The spectra shown in Figure 3 comparing MQD to States and States-TPPI applying deliberately misset pulses, with and without phase cycling for axial peak suppression, prove the axial peak suppression efficiency to be on par with the slower classical approach.

### 2.2. 3D HNCO and HCCH-TOCSY

As demonstration examples we present 3D MQD triple resonance ^1^H-^15^N-^13^CO correlation (HNCO) experiments as the proof of principle and technically more demanding 3D MQD ^1^H-^1^H correlation via ^13^C-^13^C isotropic mixing (HCCH-TOCSY) spectra of the photosynthetic protein PsbQ^26^ and of ubiqutin. The results are compared to spectra obtained using standard States-TPPI pulse sequences. We implemented the MQD scheme by modifying the standard pulse sequences^27^ as shown in the Experimental Section. In Table 1 we compare signal amplitudes, noise levels, and signal-to-noise ratios obtained by averaging over all resolved peaks in the MQD and States-TPPI HNCO and HCCH-TOCSY spectra of PsbQ and ubiquitin.

**Table 1:** Theoretical and experimental average relative signal, noise, and signal-to-noise values from standard and multiplex 3D HNCO and HCCH-TOCSY spectra

quad. scheme	rel. signal	rel. noise	rel. *S*/*N*
States TPPI	set to 1.00	set to 1.00	set to 1.00
MQD (theory)	0.56 (9/16)	0.75 (√9/√16)	0.75

Average over 102 peaks (HNCO of PsbQ)			

MQD	0.56±0.01	0.74±0.05	0.76±0.06

Average over 126 peaks (HCCH-TOCSY of ubiquitin)			

MQD	0.57±0.06	0.75±0.16	0.78±0.18

The experimentally determined ratios closely match the theoretical ones. These results corroborate that in only 56 % of the time required for conventional phase-cycled 3D experiments the MQD approach yields pure-phase spectra with the same resolution and the same per scan signal-to-noise ratio.

**Figure 2:**
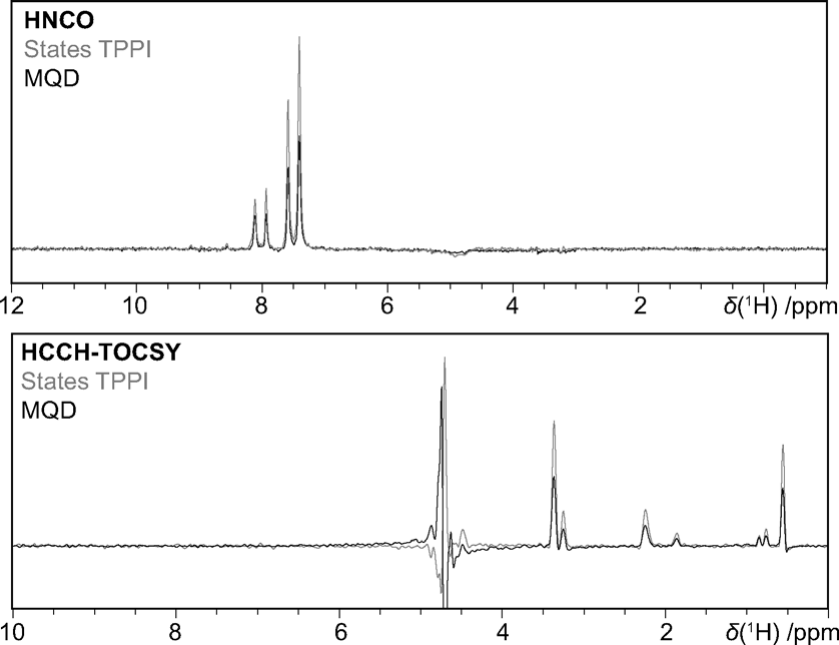
1D cross sections of MQD and standard HNCO spectra of PsbQ (*δ*_CO_=177.5 ppm, *δ*_N_=106.5 ppm) and HCCH-TOCSY spectra of ubiquitin (*δ*_C_=62.2 ppm, *δ*_H_=3.33 ppm). It can be seen that the signal ratio between the multiplex and the standard experiment closely corresponds to the theoretical value of 9/16. The solvent signal is at 4.7 ppm.

Figure 2 shows examples of 1D cross-sections of the experimentally obtained 3D spectra, which were used to determine the signal-to-noise ratios.

**Figure 3:**
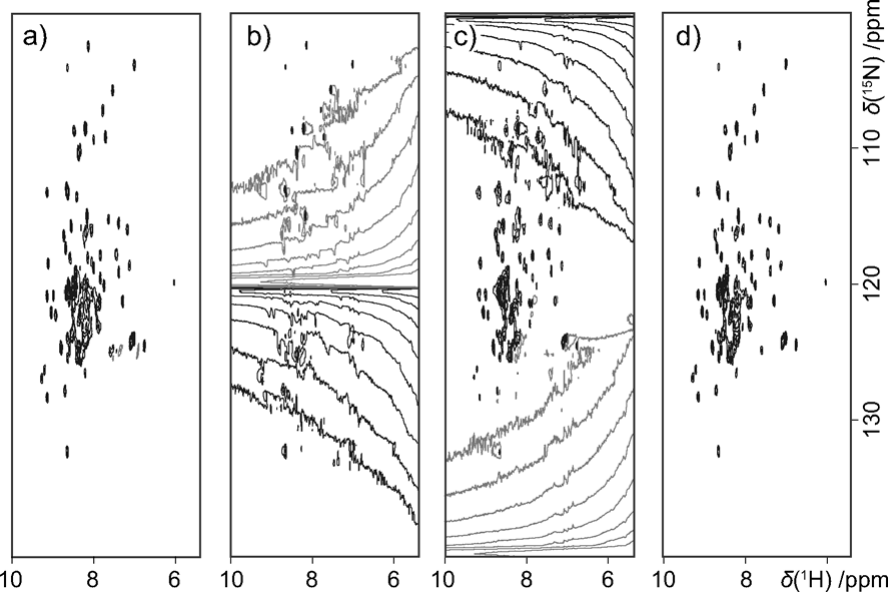
2D HNCO spectra of ubiquitin with a) MQD, b) States, without axial peak suppression phase cycle, c) States TPPI without axial peak suppression phase cycle and d) States TPPI with axial peak suppression (0°, 180°) phase cycle. The pulse lengths were misset to 90 % of the calibrated values. It can be seen that even under such unfavorable conditions the axial peak and solvent-artifact suppression efficiency of the MQD experiment (a) is equivalent to the one in the standard phase-cycled experiment (d). In all these plots, the contour levels are equal.

The reduction of the phase-cycle length raises the question, if the suppression of axial peaks is affected. To address this issue the results of axial peak suppression tests of the multiplex-quadrature scheme are shown in Figure 3. In these experiments differently quadrature-detected and phase-cycled 2D HNCO spectra of ubiquitin were obtained under suboptimal pulse conditions (all pulse lengths set to 90 % of the calibrated values).

**Figure 4:**
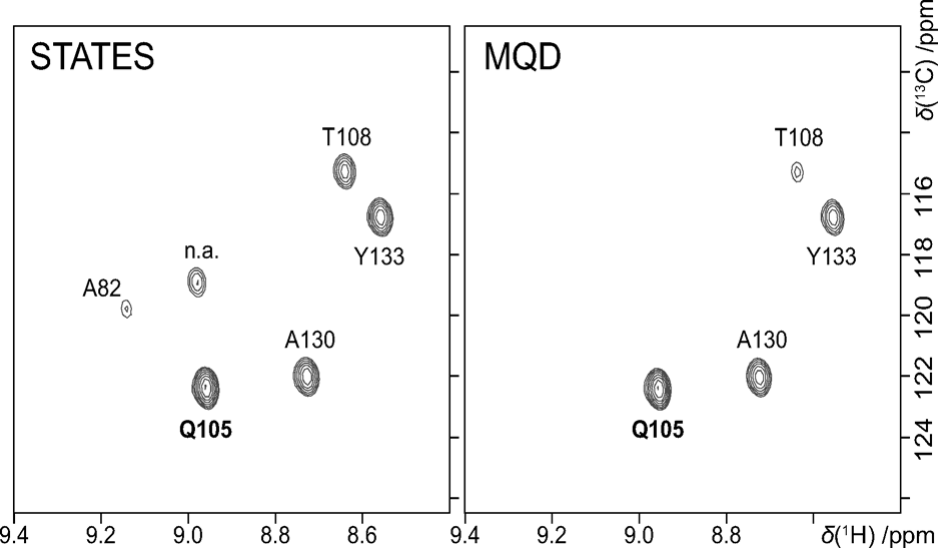
2D slices of the States-TPPI (left) and MQD (right) HNCO experiments of the protein PsbQ (including assignment of peaks; n.a. means that this peak could not be assigned).26b The 3D spectra were obtained in the same amount of time by acquiring a different number of data points in the indirect dimensions (TD1=54 and TD2=96 using the standard sequence and TD1=TD2=96 using MQD,). The MQD raw data were pre-processed as described in the Experimental Section. Then the 3D data sets were processed in TopSpin 2.1. Zero-filling in both indirect dimensions to 256 data points and squared-cosine-window functions were used.

Instead of shortening experiments at unchanged resolutions one can take the advantage of the saved time to achieve resolution enhancement. Therefore, we compare results of an MQD to a States-TPPI HNCO experiment obtained in the same amount of time. In the standard experiment 27 complex points were acquired in the carbon dimension, while in the MQD experiment 48 complex points could be recorded in the same time. In Figure 4 we show a representative region of the proton–nitrogen plane at the ^13^CO chemical shift (179.9 ppm) of residue L104. An intense cross-peak connects this C to the ^1^H^15^N pair of Q105. Six cross-peaks, four of them with similarly high amplitudes, appear in the States-TPPI HNCO due to similar CO shifts. Only the four bigger peaks appear in the same plane of the MQD HNCO experiment, and significant amplitude differences reduce assignment ambiguities substantially. This comparison corroborates the advantage of using the time saved by the multiplex approach to accommodate more evolution-time increments, thereby increasing the resolution in the indirect dimensions.

## 3. Conclusions

We introduced multiplex-quadrature detection (MQD) and implemented it on a commercial (Bruker) NMR spectrometer. The concept is generally applicable to all indirect evolution dimensions of *N*-dimensional pulsed NMR spectra and offers a time reduction of (3/4)^*N*−1^ compared to conventional spectra, while retaining resolution, axial peak suppression, and per-scan sensitivity. The processing is fully linear, requires no adjustable parameters and introduces no systematic artefacts. The method was demonstrated by a 44 % timesaving in 3D HNCO spectra of the photosynthetic protein PsbQ as well as in 3D HCCH-TOCSY spectra of ubiquitin, compared to States-TPPI-based experiments. Application of this method is recommended in cases, where the experiment duration is mainly determined by resolution rather than signal-to-noise requirements. The time saved can alternatively be used to increase the resolution in the indirect dimensions. The additional processing time for calculating linear combinations on the spectrometer’s computer is insignificant. This approach is especially beneficial for three- and higher-dimensional experiments and the combination with other time saving methods, such as sparse sampling techniques^4^ should be straightforward. Generally, we presume that many more NMR methods can be made more efficient by multiplex approaches. The immediate linear combination used in most pulse programs today is a relic of the time when computers were slow and mass storage was expensive. Storing phase-cycle steps separately should become the rule, since it allows to extract additional information just by (re-)processing. Multiplex-quadrature detection and multiplexed multiple-quantum filtered COSY^9^ are only the first steps into this direction.

## Experimental Section

3D HNCO spectra of the photosynthetic protein PsbQ^26^ (0.39 mm uniformly ^13^C,^15^N labelled PsbQ in 20 mm phosphate buffer containing 1 mm EDTA, pH 7.5, 93 %H_2_O/7 %D_2_O) as well as 3D HNCO and 3D HCCH-TOCSY spectra of uniformly 99 % ^13^C,^15^N labelled ubiquitin (1 mm) in phosphate buffer (50 mm, 95 %H_2_O/5 %D_2_O) were acquired on Bruker DRX and Avance III 500 MHz spectrometers equipped with a cryogenically cooled triple resonance (TXI) probe. Conventional probe tuning^28^ was employed and not changed between the standard and MQD experiments.

For 3D HNCO a pulse sequence27a containing a WATERGATE block^29^ and using States-TPPI^14^ for quadrature detection in both indirect dimensions was used. The multiplex approach was implemented in this pulse sequence by changing the phase cycles of the excitation pulses prior to the evolution times to be incremented in three steps of 120°. The 90° phase shifts for States-type quadrature separation were omitted. In addition each FID corresponding to a single phase-cycle step was stored separately. The analogous procedure was used in case of the 3D HCCH-TOCSY sequence. 27b The post-acquisition linear combination was implemented in C as a Bruker au-program (TopSpin 2.1), which linearly combines the original FIDs according to the virtual receiver phases [Eq. (2)] by multiplying with 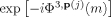. The combined data are then stored in the same manner as a double echo/anti-echo 3D data matrix, which can be processed applying the usual procedures. The modified pulse programs, the post-processing program, and additional details are available in the Supplementary Information.

## Supplementary material

Detailed facts of importance to specialist readers are published as ”Supporting Information”. Such documents are peer-reviewed, but not copy-edited or typeset. They are made available as submitted by the authors.
