# Supplementary material for: MQD—Multiplex-Quadrature Detection in Multi-Dimensional NMR
Source: Chemphyschem. 2011 Nov 16;13(1):342–6. doi: 10.1002/cphc.201100525 (PMC3298640; doi:10.1002/cphc.201100525)
Supplement: Supplementary file 1 [file cphc0013-0342-SD1.pdf]

CHEMPHYSCHEM

Supporting Information

© Copyright Wiley-VCH Verlag GmbH & Co. KGaA, 69451 Weinheim, 2011

MQD—Multiplex-Quadrature Detection in Multi-Dimensional NMR

Judith Schlagnitweit,[a] Michaela Horničáková,[a] Gerhard Zuckerstätter,[b] and Norbert Müller*[a]

cphc_201100525_sm_miscellaneous_information.pdf

HNCO:

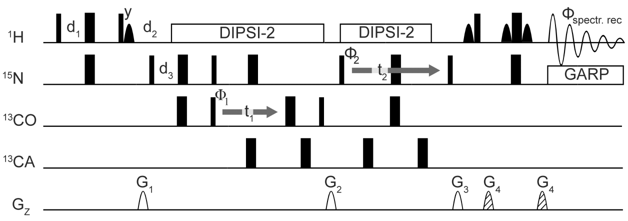

Supporting Figure 1. 3D HNCO pulse sequence containing a WATERGATE sequence[28] and using States-TPPI[14] for quadrature detection in both indirect dimensions. Wide and narrow bars represent 90- and 180-degree pulses. The delays were set to (d1=2.3ms, d2=5.5ms, d3=12ms) while the gradient values are (G1=50, G2=40, G3=60, G4=30)*0.57 Gauss/cm. The phase cycle consists of 4 steps on 2 pulses ($\Phi_1 = \pi(0,1,0,1)$, $\Phi_2 = \pi(0,0,1,1)$, $\Phi_{rec.} = \pi(0,1,1,0)$). The number of averages was four, i.e. four transients per time increment and for each quadrature step are acquired. For quadrature detection two FIDs with 90° different relative phases are acquired separately for each increment in each indirect dimension ((Φ_1+0,Φ_1+π/2), (Φ_2+0, Φ_2+π/2)). In both indirect dimensions 48 real / imaginary data points (i.e. TD1 = TD2 = 96 using Bruker's TopSpin 1.3) are acquired. In the nested multiplex version the phase cycles of the excitation pulses prior to each indirect evolution time were changed to be incremented about 120° instead of 180° (Φ_1 = 2π/3(0,1,2,0,1,2,0,1,2), Φ_1 = 2π/3(0,0,0,1,1,1,2,2,2), Φ_{rec}= 0. An additional loop in the pulse program MQDhncogpwg3D assures that the FIDs aquired for each phase cycle step are stored separately. This was done in an interleaved manner in the innermost evolution loop of the pulse program.

MQD HNCO pulse program:

```
;MQDhncogpwg3d
;avance-version (05/10/28)
;HNCO
;3D sequence with
;   inverse correlation for triple resonance using multiple
;      inept transfer steps
;
;      F1(H) -> F3(N) -> F2(C=O,t1) -> F3(N,t2) -> F1(H,t3)
;
;on/off resonance Ca and C=O pulses using shaped pulse
;phase sensitive using Multiplex Quadrature Detection(t1, t2)
;using constant time in t2
;(use parameter set HNCOGP3D)
;
;S. Grzesiek & A. Bax, J. Magn. Reson. 96, 432 - 440 (1992)
;J. Schleucher, M. Sattler & C. Griesinger, Angew. Chem. Int. Ed. 32,
;   1489-1491 (1993)
;L.E. Kay, G.Y. Xu & T. Yamazaki, J. Magn. Reson. A109, 129-133 (1994)
;
;Authors of this version:
; Judith Schlagnitweit and Norbert Müller
; Institute of Organic Chemistry
; JKU Linz, Austria
;
;$CLASS=HighRes
;$DIM=3D
;$TYPE=
;$SUBTYPE=
;$COMMENT=

prosol relations=<triple>

#include <Avance.incl>
#include <Grad.incl>
#include <Delay.incl>


"p2=p1*2"
"p22=p21*2"
"d0=3u"
"d11=30m"
"d13=4u"

"d21=5.5m"
"d23=12m"
"d26=2.3m"


"in29=in10"
"in30=in10"

"d10=d23/2-p14/2"
"d29=d23/2-p14/2-p26-d21-4u"
"d30=d23/2-p14/2"


"DELTA=d0*2+larger(p14,p22)-p14"
"DELTA1=p16+d16+d13+4u"
"DELTA2=d23-d21-p26"
"DELTA3=d21-4u"
"DELTA4=d26-p16-d16-p11-12u"
```

```
"spoff2=0"
"spoff3=0"
"spoff5=bf2*(cnst22/1000000)-o2"
"spoff8=0"

"l0=1"

aqseq 321


1 d11 ze
  d11 pl16:f3
2 d11 do:f3
3 d1 pl1:f1
  p1 ph1
  d26 pl3:f3
  (center (p2 ph1) (p22 ph1):f3 )
  d26 UNBLKGRAD
  (p1 ph2):f1

  4u pl0:f1
  (p11:sp1 ph1:r):f1
  4u
  p16:gp1
  d16

  (p21 ph3):f3
  d21 pl19:f1
  (p26 ph2):f1
  DELTA2 cpds1:f1 ph1
  (center (p14:sp3 ph1):f2 (p22 ph1):f3 )
  d23
  (p21 ph1):f3

  4u

  (p13:sp2 ph4):f2
  d0
  (center (p14:sp5 ph1):f2 (p22 ph1):f3 )
  d0
  4u
  (p14:sp3 ph1):f2
  DELTA
  (p14:sp5 ph1):f2
  4u
  (p13:sp8 ph9):f2

  4u do:f1
  (p26 ph7):f1
  4u
  p16:gp2
  d16
  (p26 ph2):f1
  20u cpds1:f1 ph1

  if "l0 <= 3"
    {
    (p21 ph8):f3
    }
  else
    {
    if "l0 <= 6"
      {
      (p21 ph18):f3
      }
    else
      {
      (p21 ph28):f3
      }
    }

  d30
  (p14:sp5 ph1):f2
  d30
  (center (p14:sp3 ph1):f2 (p22 ph1):f3 )
  d10
  (p14:sp5 ph1):f2
  d29
  4u do:f1
  (p26 ph7):f1
  d21
  (p21 ph1):f3

  p16:gp3
  d16 pl0:f1
  (p11:sp1 ph6):f1
  4u
  4u pl1:f1

  (p1 ph1)
  4u
  p16:gp4
  d16
  DELTA4 pl0:f1
  (p11:sp1 ph6):f1
  4u
  4u pl1:f1
  (center (p2 ph1) (p22 ph1):f3 )
  4u pl0:f1
  (p11:sp1 ph6):f1
  4u
  DELTA4
  p16:gp4
  d16 pl16:f3
  4u BLKGRAD
```

```
  go=2 ph31 ph30:r cpd3:f3
  d11 do:f3 mc #0 to 2
    F1QF(rd10 & rd29 & rd30 & id0)
          F2I(ip4*4 & iu0, 9)
          F2QF(ru0 & id10 & id29 & dd30)
;     F1PH(rd10 & rd29 & rd30 & ip4, id0)
;     F2EA(igrad EA & ip6*2, id10 & id29 & dd30)
exit


ph1=0
ph2=1
ph3=0
ph4=(12) 0
ph5=0
ph6=2
ph7=3
ph8=(12) 0
ph18=(12) 4
ph28=(12) 8
ph9=0
ph30=0
ph31=0


;pl0 : 120dB
;pl1 : f1 channel - power level for pulse (default)
;pl3 : f3 channel - power level for pulse (default)
;pl16: f3 channel - power level for CPD/BB decoupling
;pl19: f1 channel - power level for CPD/BB decoupling
;sp1: f1 channel - shaped pulse  90 degree  (H2O on resonance)
;sp2: f2 channel - shaped pulse  90 degree  (C=O on resonance)
;sp3: f2 channel - shaped pulse 180 degree  (C=O on resonance)
;sp5: f2 channel - shaped pulse 180 degree  (Ca off resonance)
;sp8: f2 channel - shaped pulse  90 degree  (C=O on resonance)
;                   for time reversed pulse
;p1 : f1 channel -  90 degree high power pulse
;p2 : f1 channel - 180 degree high power pulse
;p11: f1 channel -  90 degree shaped pulse             [2 msec]
;p13: f2 channel -  90 degree shaped pulse
;p14: f2 channel - 180 degree shaped pulse
;p16: homospoil/gradient pulse                         [1 msec]
;p21: f3 channel -  90 degree high power pulse
;p22: f3 channel - 180 degree high power pulse
;p26: f1 channel -  90 degree pulse at pl19
;d0 : incremented delay (F1 in 3D)                     [3 usec]
;d1 : relaxation delay; 1-5 * T1
;d10: incremented delay (F2 in 3D) =  d23/2-p14/2
;d11: delay for disk I/O                               [30 msec]
;d13: short delay                                      [4 usec]
;d16: delay for homospoil/gradient recovery
;d21: 1/(2J(NH)                                        [5.5 msec]
;d23: 1/(4J(NCO)                                       [12 msec]
;d26: 1/(4J'(NH)                                       [2.3 msec]
;d29: incremented delay (F2 in 3D) = d23/2-p14/2-p26-d21-4u
;d30: decremented delay (F2 in 3D) = d23/2-p14/2
;cnst21: CO chemical shift (offset, in ppm)
;cnst22: Calpha chemical shift (offset, in ppm)
;o2p: CO chemical shift (cnst21)
;in0: 1/(2 * SW(CO)) =  DW(CO)
;nd0: 2
;in10: 1/(4 * SW(N)) = (1/2) DW(N)
;nd10: 4
;in29: = in10
;in30: = in10
;NS: 8 * n
;DS: >= 16
;td1: number of experiments in F1
;td2: number of experiments in F2       td2 max = 2 * d30 / in30
;cpds1: decoupling according to sequence defined by cpdprg1
;cpd3: decoupling according to sequence defined by cpdprg3
;pcpd1: f1 channel - 90 degree pulse for decoupling sequence
;pcpd3: f3 channel - 90 degree pulse for decoupling sequence

;FnMODE: QF in F1
;FnMODE: QF in F2


;use gradient ratio:    gp 1 : gp 2 : gp 3 : gp 4
;                         50 :   40 :   60 :   30

;for z-only gradients:
;gpz1: 50%
;gpz2: 40%
;gpz3: 60%
;gpz4: 30%

;use gradient files:
;gpnam1: SINE.100
;gpnam2: SINE.100
;gpnam3: SINE.100
;gpnam4: SINE.100
;gpnam5: SINE.100


;processing
;use au-program "MQD_F2" to generate the raw data set for processing!
;then use echo-antiecho in both dimensions for processing !!!!
```

HCCH-TOCSY:

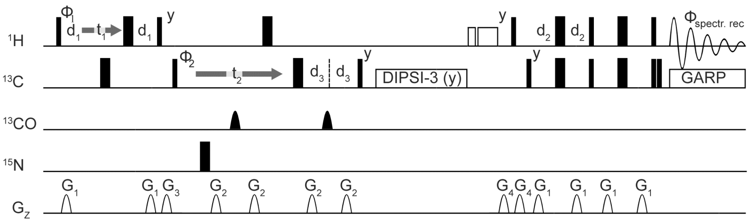

Supporting Figure 2. 3D HCCH-TOCSY pulse sequence[27b] using States-TPPI[14] for quadrature detection in both indirect dimensions. Wide and narrow bars represent 90- and 180-degree pulses. The delays were set to (d1=1.6ms, d2=1.1ms, d3=0.475ms) while the gradient values are (G1=16, G2=16, G3=30, G4=60)*0.57 Gauss/cm. The phase cycle consists of 4 steps on 2 pulses ($\Phi_1 = \pi(0,1,0,1)$, $\Phi_2 = \pi(0,0,1,1)$, $\Phi_{rec.} = \pi(0,1,1,0)$). The number of averages was four, i.e. four transients per time increment and for each quadrature step are acquired. For quadrature detection two FIDs with 90° different relative phases are acquired separately for each increment in each indirect dimension ((Φ_1+0,Φ_1+π/2), (Φ_2+0, Φ_2+π/2)). In both indirect dimensions 48 real / imaginary data points (i.e. TD1 = TD2 = 96 using Bruker's TopSpin 1.3) are acquired. In the nested multiplex version the phase cycles of the excitation pulses prior to each indirect evolution time were changed to be incremented about 120° instead of 180° ($\Phi_1 = 2\pi/3(0,1,2,0,1,2,0,1,2)$, $\Phi_1 = 2\pi/3(0,0,0,1,1,1,2,2,2)$, Φ_{rec}= 0. An additional loop in the pulse program assures that the FIDs aquired for each phase cycle step are stored separately. This was done in an interleaved manner in the innermost evolution loop of the pulse program MQDhcchdigp3d.

MQD HCCH-TOCSY pulse program:

```
;MQDhcchdigp3d
;avance-version (11/09/12)
;HCCH-TOCSY
;3D sequence with
;   inverse correlation using multiple inept transfer and
;      C-C DIPSI3 spinlock
;
;      F1(H,t1) -> F2(C,t2) -> F2(C') -> F1(H',t3)
;
;off resonance C=O pulse using shaped pulse
;phase sensitive using Multiplex Quadrature Detection(t1, t2)

;spinlock during z-filter
;(use parameterset HCCHDIGP3D)
;
;(L.E. Kay, G.Y. Xu, A.U. Singer, D.R. Muhandiram & J. D. Forman-Kay
;   J. Magn. Reson. B 101, 333 - 337 (1993))
;
; Authors of this version:
; Judith Schlagnitweit and Norbert Müller
; Institute of Organic Chemistry
; JKU Linz, Austria
;

;$CLASS=HighRes
;$DIM=3D
;$TYPE=
;$SUBTYPE=
;$COMMENT=

prosol relations=<triple>

#include <Avance.incl>
#include <Grad.incl>
#include <Delay.incl>


"p2=p1*2"
"p4=p3*2"
"p22=p21*2"
"d11=30m"
"d12=20u"

"d4=1.6m"                        ;tau a
"d21=1.1m"                                 ;tau c
"d23=475u"                                 ;tau b


"p16=500u"
"p19=2m"
"p29=300u"
"p30=5m"
"p31=4.4m"

"d16=150u"


"d0=3u"
"d10=3u"

"in0=inf1/2"
"in10=inf2/2"


"DELTA1=d4-p16-d16-4u"
"DELTA2=d4-p16-d16-4u+d0*2+p4"
"DELTA3=d23-p29-d16"
"DELTA4=p22+p2+d10*2+4u"
```

```
"DELTA5=d21-p16-d16-4u"
"DELTA6=d4-p16-d16-p3*2-7u+p1"


"d31=(p9*54.33*4)*l1"


"spoff5=bf2*(cnst21/1000000)-o2"

"l0=0"

aqseq 321


1 d11 ze
  d31 pl12:f2
2 d11 do:f2
3 d1
  50u UNBLKGRAD
  d12 pl1:f1

  20u
  "cnst30=(l0 %3)*120"
  20u
  "cnst31=((trunc(l0/3)) %3)*120"

  3m ip3+cnst30
  3m ip4+cnst31

  (p1 ph3)
  4u
  p16:gp1
  d16
  DELTA1 pl2:f2
  d0
  (p4 ph1):f2
  d0
  (p2 ph1)
  4u
  p16:gp1
  d16
  DELTA2 pl3:f3
  (p1 ph2)

  p19:gp3
  d16

  (p3 ph4):f2
  d10
  (p22 ph1):f3
  4u
  p29:gp2
  d16
  DELTA3 pl0:f2
  (p14:sp5 ph1):f2
  4u
  p29:gp2
  d16
  DELTA3 pl2:f2
  p2 ph1
  d10
  (p4 ph1):f2
  DELTA4
  p29:gp2
  d16
  DELTA3
  (p14:sp5 ph1):f2
  4u
  p29:gp2
  d16
  DELTA3 pl2:f2
  (p3 ph2):f2
  4u
  d12 pl15:f2
                                                            ;begin DIPSI3
9 (p9*2.722 ph7):f2
  (p9*4.389 ph9):f2
  (p9*2.778 ph7):f2
  (p9*3.056 ph9):f2
  (p9*0.333 ph7):f2
  (p9*2.556 ph9):f2
  (p9*4.000 ph7):f2
  (p9*2.722 ph9):f2
  (p9*4.111 ph7):f2
  (p9*3.778 ph9):f2
  (p9*3.889 ph7):f2
  (p9*2.889 ph9):f2
  (p9*3.000 ph7):f2
  (p9*0.333 ph9):f2
  (p9*2.500 ph7):f2
  (p9*4.050 ph9):f2
  (p9*2.830 ph7):f2
  (p9*4.389 ph9):f2
  (p9*2.722 ph9):f2
  (p9*4.389 ph7):f2
  (p9*2.778 ph9):f2
  (p9*3.056 ph7):f2
  (p9*0.333 ph9):f2
  (p9*2.556 ph7):f2
  (p9*4.000 ph9):f2
  (p9*2.722 ph7):f2
  (p9*4.111 ph9):f2
  (p9*3.778 ph7):f2
  (p9*3.889 ph9):f2
  (p9*2.889 ph7):f2
  (p9*3.000 ph9):f2
```

```
  (p9*0.333 ph7):f2
  (p9*2.500 ph9):f2
  (p9*4.050 ph7):f2
  (p9*2.830 ph9):f2
  (p9*4.389 ph7):f2
  (p9*2.722 ph9):f2
  (p9*4.389 ph7):f2
  (p9*2.778 ph9):f2
  (p9*3.056 ph7):f2
  (p9*0.333 ph9):f2
  (p9*2.556 ph7):f2
  (p9*4.000 ph9):f2
  (p9*2.722 ph7):f2
  (p9*4.111 ph9):f2
  (p9*3.778 ph7):f2
  (p9*3.889 ph9):f2
  (p9*2.889 ph7):f2
  (p9*3.000 ph9):f2
  (p9*0.333 ph7):f2
  (p9*2.500 ph9):f2
  (p9*4.050 ph7):f2
  (p9*2.830 ph9):f2
  (p9*4.389 ph7):f2
  (p9*2.722 ph7):f2
  (p9*4.389 ph9):f2
  (p9*2.778 ph7):f2
  (p9*3.056 ph9):f2
  (p9*0.333 ph7):f2
  (p9*2.556 ph9):f2
  (p9*4.000 ph7):f2
  (p9*2.722 ph9):f2
  (p9*4.111 ph7):f2
  (p9*3.778 ph9):f2
  (p9*3.889 ph7):f2
  (p9*2.889 ph9):f2
  (p9*3.000 ph7):f2
  (p9*0.333 ph9):f2
  (p9*2.500 ph7):f2
  (p9*4.050 ph9):f2
  (p9*2.830 ph7):f2
  (p9*4.389 ph9):f2
  lo to 9 times l1
                                                          ;end DIPSI3
  d12 pl10:f1
  (p17 ph1)
  (p17*2 ph2)
  4u
  p30:gp4
  d16 pl1:f1
  (p1 ph1)
  4u
  p31:gp4
  d16 pl2:f2

  (p3 ph2):f2
  4u
  p16:gp1
  d16
  DELTA5
  (center (p2 ph1) (p4 ph1):f2 )
  4u
  p16:gp1
  d16
  DELTA5
  (center (p1 ph1) (p3 ph1):f2 )

  4u
  p16:gp1
  d16
  DELTA1
  (center (p2 ph1) (p4 ph1):f2 )
  4u
  p16:gp1
  d16
  DELTA6
  (center (p1 ph1) (p3 ph1 3u p3 ph5):f2 )
  4u pl12:f2
  4u BLKGRAD
  go=2 ph31 cpd2:f2
  d11 do:f2 mc #0 to 2
     F1QF(caldel(d0, +in0))
     F2I(iu0, 9)
     F2QF(caldel(d10, +in10))
exit


ph1=0
ph2=1
ph3=0
ph4=0
ph5=0
ph7=1
ph9=3
ph31=0


;pl0 : 0W
;pl1 : f1 channel - power level for pulse (default)
;pl2 : f2 channel - power level for pulse (default)
;pl3 : f3 channel - power level for pulse (default)
;pl10: f1 channel - power level for TOCSY-spinlock (trim pulse)
;pl12: f2 channel - power level for CPD/BB decoupling
;pl15: f2 channel - power level for TOCSY-spinlock
;sp5: f2 channel - shaped pulse 180 degree   (C=O off resonance)
;p1 : f1 channel -  90 degree high power pulse
;p2 : f1 channel - 180 degree high power pulse
```

```
;p3 : f2 channel -  90 degree high power pulse
;p4 : f2 channel - 180 degree high power pulse
;p9 : f2 channel -  90 degree low power pulse
;p14: f2 channel - 180 degree shaped pulse
;p16: homospoil/gradient pulse                         [500 usec]
;p17: f1 channel - trim pulse                          [2.5 msec]
;p19: gradient pulse 2                                 [2 msec]
;p22: f3 channel - 180 degree high power pulse
;p29: gradient pulse 3                                 [300 usec]
;p30: gradient pulse 4                                 [5 msec]
;p31: gradient pulse 5                                 [4.4 msec]
;d0 : incremented delay (F1 in 3D)                     [3 usec]
;d1 : relaxation delay; 1-5 * T1
;d4 : 1/(4J(CH)) - tau a                               [1.6 msec]
;d10: incremented delay (F2 in 3D)                     [3 usec]
;d11: delay for disk I/O                               [30 msec]
;d12: delay for power switching                        [20 usec]
;d16: delay for homospoil/gradient recovery
;d21: 1/(6J'(CH)) - tau c                              [1.1 msec]
;d23: tau b                                            [475 usec]
;d31: length of DIPSI-3 cycle as executed = (p9*54.33*4)*l1
;cnst21: CO chemical shift (offset, in ppm)
;cnst23: Caliphatic chemical shift (offset, in ppm)
;o2p: Caliphatic chemical shift (cnst23)
;l1: loop for DIPSI cycle:
;       mixing time = ((p9*54.33*4) * l1)              [12 msec]
;inf1: 1/SW(Hali) = 2 * DW(Hali)
;inf2: 1/SW(C) = 2 * DW(C)
;in0: 1/(2 * SW(Hali)) = DW(Hali)
;nd0: 2
;in10: 1/(2 * SW(C)) = DW(C)
;nd10: 2
;NS: 8 * n
;DS: 32
;td1: number of experiments in F1
;td2: number of experiments in F2
;FnMODE: QF in F1
;FnMODE: QF in F2
;cpd2: decoupling according to sequence defined by cpdprg2
;pcpd2: f2 channel - 90 degree pulse for decoupling sequence


;use gradient ratio:    gp 1 : gp 2 : gp 3 : gp 4
;                         16 :   16 :   30 :   60

;for z-only gradients:
;gpz1: 16%
;gpz2: 16%
;gpz3: 30%
;gpz4: 60%

;use gradient files:
;gpnam1: SMSQ10.50
;gpnam2: SMSQ10.50
;gpnam3: SMSQ10.100
;gpnam4: SMSQ10.100

;Processing
;use au-program "MQD_F2" to generate the raw data set for processing!
;then use echo-antiecho in both dimensions for processing !!!!
```

MQD pre-processing au program:

```
/****************************************************************/
/*      MQD_F2                                                  */
/****************************************************************/
/*      Short Description :                                     */
/*      AU program used for Multiplex Quad. Det. to calculate   */
/*      a 3D echo-/anti-echo data set out of the interleaved    */
/*      raw data set.                                           */
/****************************************************************/
/*      Description/Usage :                                     */
/*      The AU program is suitable for raw data sets obtained   */
/*      using the pulse program "MQDhncogpwg3d"                 */
/*      The interleaved FIDs are linearly combined, re-sorted   */
/*      and stored in the desired expno.                        */
/*      This new data set can be processed in the same way      */
/*      as a common echo- / anti-echo 3D data set.              */
/*          The program is based on the Bruker au-program       */
/*          "split" written by Wolfgang Bermel                  */
/*          Written for Topspin2.1.                             */
/****************************************************************/
/*                 Authors:                                     */
/*                 Judith Schlagnitweit and Norbert Müller      */
/*                 Institute of Organic Chemistry               */
/*                 JKU Linz, Austria                            */
/****************************************************************/

#define MAXSIZE 196608

  char infile[PATH_MAX], outfile1[PATH_MAX], outfile2[PATH_MAX];
  char infile10[PATH_MAX], infile20[PATH_MAX], outfile10[PATH_MAX];
  char path[PATH_MAX + 64];

  int *row1, *row2, *row3, *row4, *row5;
  int *row6, *row7, *row8, *row9;
  int row11[MAXSIZE], row12[MAXSIZE];
  int td, td1, td2, tds, td1s, td2s;
  int i, j;
```

```
  int nexpno, newexpno, byteorder, parmode, aqseq = -1;
  int split;

  float phase1_1, phase2_1, phase3_1, phase4_1, phase5_1, phase6_1, phase7_1, phase8_1, phase9_1;
  float phase1_2, phase2_2, phase3_2, phase4_2, phase5_2, phase6_2, phase7_2, phase8_2, phase9_2;
  float phase1_3, phase2_3, phase3_3, phase4_3, phase5_3, phase6_3, phase7_3, phase8_3, phase9_3;
  float phase1_4, phase2_4, phase3_4, phase4_4, phase5_4, phase6_4, phase7_4, phase8_4, phase9_4;
  float arg1_1, arg2_1, arg3_1, arg4_1, arg5_1, arg6_1, arg7_1, arg8_1, arg9_1;
  float arg1_2, arg2_2, arg3_2, arg4_2, arg5_2, arg6_2, arg7_2, arg8_2, arg9_2;
  float arg1_3, arg2_3, arg3_3, arg4_3, arg5_3, arg6_3, arg7_3, arg8_3, arg9_3;
  float arg1_4, arg2_4, arg3_4, arg4_4, arg5_4, arg6_4, arg7_4, arg8_4, arg9_4;

  FILE *fpin, *fpout1, *fpout2;
  FILE *fpin10, *fpin20, *fpout10;

  char* endpath;

/***** define coefficients *****/

  phase1_1 =   0.0;
  phase2_1 =   240.0;
  phase3_1 =   120.0;
  phase4_1 =   240.0;
  phase5_1 =   120.0;
  phase6_1 =   0.0;
  phase7_1 =   120.0;
  phase8_1 =   0.0;
  phase9_1 =   240.0;

  phase1_2 =   0.0;
  phase2_2 =   240.0;
  phase3_2 =   120.0;
  phase4_2 =   120.0;
  phase5_2 =   0.0;
  phase6_2 =   240.0;
  phase7_2 =   240.0;
  phase8_2 =   120.0;
  phase9_2 =   0.0;

  phase1_3 =   0.0;
  phase2_3 =   120.0;
  phase3_3 =   240.0;
  phase4_3 =   240.0;
  phase5_3 =   0.0;
  phase6_3 =   120.0;
  phase7_3 =   120.0;
  phase8_3 =   240.0;
  phase9_3 =   0.0;

  phase1_4 =   0.0;
  phase2_4 =   120.0;
  phase3_4 =   240.0;
  phase4_4 =   120.0;
  phase5_4 =   240.0;
  phase6_4 =   0.0;
  phase7_4 =   240.0;
  phase8_4 =   0.0;
  phase9_4 =   120.0;


  arg1_1 = phase1_1 * M_PI / 180.0;
  arg2_1 = phase2_1 * M_PI / 180.0;
  arg3_1 = phase3_1 * M_PI / 180.0;
  arg4_1 = phase4_1 * M_PI / 180.0;
  arg5_1 = phase5_1 * M_PI / 180.0;
  arg6_1 = phase6_1 * M_PI / 180.0;
  arg7_1 = phase7_1 * M_PI / 180.0;
  arg8_1 = phase8_1 * M_PI / 180.0;
  arg9_1 = phase9_1 * M_PI / 180.0;

  arg1_2 = phase1_2 * M_PI / 180.0;
  arg2_2 = phase2_2 * M_PI / 180.0;
  arg3_2 = phase3_2 * M_PI / 180.0;
  arg4_2 = phase4_2 * M_PI / 180.0;
  arg5_2 = phase5_2 * M_PI / 180.0;
  arg6_2 = phase6_2 * M_PI / 180.0;
  arg7_2 = phase7_2 * M_PI / 180.0;
  arg8_2 = phase8_2 * M_PI / 180.0;
  arg9_2 = phase9_2 * M_PI / 180.0;

  arg1_3 = phase1_3 * M_PI / 180.0;
  arg2_3 = phase2_3 * M_PI / 180.0;
  arg3_3 = phase3_3 * M_PI / 180.0;
  arg4_3 = phase4_3 * M_PI / 180.0;
  arg5_3 = phase5_3 * M_PI / 180.0;
  arg6_3 = phase6_3 * M_PI / 180.0;
  arg7_3 = phase7_3 * M_PI / 180.0;
  arg8_3 = phase8_3 * M_PI / 180.0;
  arg9_3 = phase9_3 * M_PI / 180.0;

  arg1_4 = phase1_4 * M_PI / 180.0;
  arg2_4 = phase2_4 * M_PI / 180.0;
  arg3_4 = phase3_4 * M_PI / 180.0;
  arg4_4 = phase4_4 * M_PI / 180.0;
  arg5_4 = phase5_4 * M_PI / 180.0;
  arg6_4 = phase6_4 * M_PI / 180.0;
  arg7_4 = phase7_4 * M_PI / 180.0;
  arg8_4 = phase8_4 * M_PI / 180.0;
  arg9_4 = phase9_4 * M_PI / 180.0;

  /***** get dataset and parameters *****/

  FETCHPAR("PARMODE", &parmode)

  if (parmode != 2)
    STOPMSG("Program is only suitable for 3D data")
```

```
  FETCHPARS("BYTORDA", &byteorder)
  FETCHPARS("TD", &tds)

  td = ((tds + 255) / 256) * 256;

  FETCHPAR3("TD", &td1)
  FETCHPAR3S("TD", &td1s)

  FETCHPAR1("TD", &td2)
  FETCHPAR1S("TD", &td2s)

  /***** get input *****/

  split = 9.0;

  nexpno = 99998;

  GETINT("Enter EXPNO to store new dataset:", newexpno)

  /***** check files *****/

  WRAPARAM(nexpno)
  ERRORABORT
  WRAPARAM(nexpno+1)
  ERRORABORT

  endpath = path + sprintf(path, "%s/data/%s/nmr/%s/", disk, user, name);
  (void)sprintf(infile, "%s%d/ser", path, expno);
  fpin = fopen(infile, "rb");

  (void)sprintf(outfile1, "%s%d/ser", path, nexpno);
          fpout1 = fopen(outfile1, "wb");
          (void)sprintf(outfile2, "%s%d/ser", path, nexpno+1);
  fpout2 = fopen(outfile2, "wb");


  /***** allocate memory *****/

  row1 = (int*)malloc(td * split * sizeof(int));
  row2 = row1 + td;
  row3 = row2 + td;
  row4 = row3 + td;
  row5 = row4 + td;
  row6 = row5 + td;
  row7 = row6 + td;
  row8 = row7 + td;
  row9 = row8 + td;

/***** split and linearly combine *****/

  Show_status("splitting data");

  for (i = 0; i < (td1s * td2s) / 9 ; i++)
  {
    if (fread(row1, sizeof(int), td, fpin) != (size_t)(td))
      STOPMSG("read failed")
    if (fread(row2, sizeof(int), td, fpin) != (size_t)(td))
      STOPMSG("read failed")
    if (fread(row3, sizeof(int), td, fpin) != (size_t)(td))
      STOPMSG("read failed")
    if (fread(row4, sizeof(int), td, fpin) != (size_t)(td))
      STOPMSG("read failed")
    if (fread(row5, sizeof(int), td, fpin) != (size_t)(td))
      STOPMSG("read failed")
    if (fread(row6, sizeof(int), td, fpin) != (size_t)(td))
      STOPMSG("read failed")
    if (fread(row7, sizeof(int), td, fpin) != (size_t)(td))
      STOPMSG("read failed")
    if (fread(row8, sizeof(int), td, fpin) != (size_t)(td))
      STOPMSG("read failed")
    if (fread(row9, sizeof(int), td, fpin) != (size_t)(td))
      STOPMSG("read failed")

    local_swap4(row1, td * sizeof(int), byteorder);
    local_swap4(row2, td * sizeof(int), byteorder);
    local_swap4(row3, td * sizeof(int), byteorder);
    local_swap4(row4, td * sizeof(int), byteorder);
    local_swap4(row5, td * sizeof(int), byteorder);
    local_swap4(row6, td * sizeof(int), byteorder);
    local_swap4(row7, td * sizeof(int), byteorder);
    local_swap4(row8, td * sizeof(int), byteorder);
    local_swap4(row9, td * sizeof(int), byteorder);

      int j;
      for (j = 0; j < td; j += 2)
      {
                              int row_r1_1 = (row1[j] * cos(arg1_1) - row1[j+1] * sin(arg1_1));
                              int row_i1_1 = (row1[j+1] * cos(arg1_1) + row1[j] * sin(arg1_1));
                              int row_r2_1 = (row2[j] * cos(arg2_1) - row2[j+1] * sin(arg2_1));
                              int row_i2_1 = (row2[j+1] * cos(arg2_1) + row2[j] * sin(arg2_1));
                              int row_r3_1 = (row3[j] * cos(arg3_1) - row3[j+1] * sin(arg3_1));
                              int row_i3_1 = (row3[j+1] * cos(arg3_1) + row3[j] * sin(arg3_1));
                              int row_r4_1 = (row4[j] * cos(arg4_1) - row4[j+1] * sin(arg4_1));
                              int row_i4_1 = (row4[j+1] * cos(arg4_1) + row4[j] * sin(arg4_1));
                              int row_r5_1 = (row5[j] * cos(arg5_1) - row5[j+1] * sin(arg5_1));
                              int row_i5_1 = (row5[j+1] * cos(arg5_1) + row5[j] * sin(arg5_1));
                              int row_r6_1 = (row6[j] * cos(arg6_1) - row6[j+1] * sin(arg6_1));
                              int row_i6_1 = (row6[j+1] * cos(arg6_1) + row6[j] * sin(arg6_1));
                              int row_r7_1 = (row7[j] * cos(arg7_1) - row7[j+1] * sin(arg7_1));
                              int row_i7_1 = (row7[j+1] * cos(arg7_1) + row7[j] * sin(arg7_1));
                              int row_r8_1 = (row8[j] * cos(arg8_1) - row8[j+1] * sin(arg8_1));
                              int row_i8_1 = (row8[j+1] * cos(arg8_1) + row8[j] * sin(arg8_1));
                              int row_r9_1 = (row9[j] * cos(arg9_1) - row9[j+1] * sin(arg9_1));
                              int row_i9_1 = (row9[j+1] * cos(arg9_1) + row9[j] * sin(arg9_1));
```

```
                        int row_r1_2 = (row1[j] * cos(arg1_2) - row1[j+1] * sin(arg1_2));
                        int row_i1_2 = (row1[j+1] * cos(arg1_2) + row1[j] * sin(arg1_2));
                        int row_r2_2 = (row2[j] * cos(arg2_2) - row2[j+1] * sin(arg2_2));
                        int row_i2_2 = (row2[j+1] * cos(arg2_2) + row2[j] * sin(arg2_2));
                        int row_r3_2 = (row3[j] * cos(arg3_2) - row3[j+1] * sin(arg3_2));
                        int row_i3_2 = (row3[j+1] * cos(arg3_2) + row3[j] * sin(arg3_2));
                        int row_r4_2 = (row4[j] * cos(arg4_2) - row4[j+1] * sin(arg4_2));
                        int row_i4_2 = (row4[j+1] * cos(arg4_2) + row4[j] * sin(arg4_2));
                        int row_r5_2 = (row5[j] * cos(arg5_2) - row5[j+1] * sin(arg5_2));
                        int row_i5_2 = (row5[j+1] * cos(arg5_2) + row5[j] * sin(arg5_2));
                        int row_r6_2 = (row6[j] * cos(arg6_2) - row6[j+1] * sin(arg6_2));
                        int row_i6_2 = (row6[j+1] * cos(arg6_2) + row6[j] * sin(arg6_2));
                        int row_r7_2 = (row7[j] * cos(arg7_2) - row7[j+1] * sin(arg7_2));
                        int row_i7_2 = (row7[j+1] * cos(arg7_2) + row7[j] * sin(arg7_2));
                        int row_r8_2 = (row8[j] * cos(arg8_2) - row8[j+1] * sin(arg8_2));
                        int row_i8_2 = (row8[j+1] * cos(arg8_2) + row8[j] * sin(arg8_2));
                        int row_r9_2 = (row9[j] * cos(arg9_2) - row9[j+1] * sin(arg9_2));
                        int row_i9_2 = (row9[j+1] * cos(arg9_2) + row9[j] * sin(arg9_2));

                        int row_r1_3 = (row1[j] * cos(arg1_3) - row1[j+1] * sin(arg1_3));
                        int row_i1_3 = (row1[j+1] * cos(arg1_3) + row1[j] * sin(arg1_3));
                        int row_r2_3 = (row2[j] * cos(arg2_3) - row2[j+1] * sin(arg2_3));
                        int row_i2_3 = (row2[j+1] * cos(arg2_3) + row2[j] * sin(arg2_3));
                        int row_r3_3 = (row3[j] * cos(arg3_3) - row3[j+1] * sin(arg3_3));
                        int row_i3_3 = (row3[j+1] * cos(arg3_3) + row3[j] * sin(arg3_3));
                        int row_r4_3 = (row4[j] * cos(arg4_3) - row4[j+1] * sin(arg4_3));
                        int row_i4_3 = (row4[j+1] * cos(arg4_3) + row4[j] * sin(arg4_3));
                        int row_r5_3 = (row5[j] * cos(arg5_3) - row5[j+1] * sin(arg5_3));
                        int row_i5_3 = (row5[j+1] * cos(arg5_3) + row5[j] * sin(arg5_3));
                        int row_r6_3 = (row6[j] * cos(arg6_3) - row6[j+1] * sin(arg6_3));
                        int row_i6_3 = (row6[j+1] * cos(arg6_3) + row6[j] * sin(arg6_3));
                        int row_r7_3 = (row7[j] * cos(arg7_3) - row7[j+1] * sin(arg7_3));
                        int row_i7_3 = (row7[j+1] * cos(arg7_3) + row7[j] * sin(arg7_3));
                        int row_r8_3 = (row8[j] * cos(arg8_3) - row8[j+1] * sin(arg8_3));
                        int row_i8_3 = (row8[j+1] * cos(arg8_3) + row8[j] * sin(arg8_3));
                        int row_r9_3 = (row9[j] * cos(arg9_3) - row9[j+1] * sin(arg9_3));
                        int row_i9_3 = (row9[j+1] * cos(arg9_3) + row9[j] * sin(arg9_3));

                        int row_r1_4 = (row1[j] * cos(arg1_4) - row1[j+1] * sin(arg1_4));
                        int row_i1_4 = (row1[j+1] * cos(arg1_4) + row1[j] * sin(arg1_4));
                        int row_r2_4 = (row2[j] * cos(arg2_4) - row2[j+1] * sin(arg2_4));
                        int row_i2_4 = (row2[j+1] * cos(arg2_4) + row2[j] * sin(arg2_4));
                        int row_r3_4 = (row3[j] * cos(arg3_4) - row3[j+1] * sin(arg3_4));
                        int row_i3_4 = (row3[j+1] * cos(arg3_4) + row3[j] * sin(arg3_4));
                        int row_r4_4 = (row4[j] * cos(arg4_4) - row4[j+1] * sin(arg4_4));
                        int row_i4_4 = (row4[j+1] * cos(arg4_4) + row4[j] * sin(arg4_4));
                        int row_r5_4 = (row5[j] * cos(arg5_4) - row5[j+1] * sin(arg5_4));
                        int row_i5_4 = (row5[j+1] * cos(arg5_4) + row5[j] * sin(arg5_4));
                        int row_r6_4 = (row6[j] * cos(arg6_4) - row6[j+1] * sin(arg6_4));
                        int row_i6_4 = (row6[j+1] * cos(arg6_4) + row6[j] * sin(arg6_4));
                        int row_r7_4 = (row7[j] * cos(arg7_4) - row7[j+1] * sin(arg7_4));
                        int row_i7_4 = (row7[j+1] * cos(arg7_4) + row7[j] * sin(arg7_4));
                        int row_r8_4 = (row8[j] * cos(arg8_4) - row8[j+1] * sin(arg8_4));
                        int row_i8_4 = (row8[j+1] * cos(arg8_4) + row8[j] * sin(arg8_4));
                        int row_r9_4 = (row9[j] * cos(arg9_4) - row9[j+1] * sin(arg9_4));
                        int row_i9_4 = (row9[j+1] * cos(arg9_4) + row9[j] * sin(arg9_4));

                        row1[j]   =  row_r1_1 + row_r2_1 + row_r3_1 + row_r4_1 + row_r5_1 + row_r6_1 + row_r7_1 + row_r8_1 + row_r9_1;
                        row1[j+1] =  row_i1_1 + row_i2_1 + row_i3_1 + row_i4_1 + row_i5_1 + row_i6_1 + row_i7_1 + row_i8_1 + row_i9_1;
                        row2[j]   =  row_r1_2 + row_r2_2 + row_r3_2 + row_r4_2 + row_r5_2 + row_r6_2 + row_r7_2 + row_r8_2 + row_r9_2;
                        row2[j+1] =  row_i1_2 + row_i2_2 + row_i3_2 + row_i4_2 + row_i5_2 + row_i6_2 + row_i7_2 + row_i8_2 + row_i9_2;
                        row3[j]   =  row_r1_3 + row_r2_3 + row_r3_3 + row_r4_3 + row_r5_3 + row_r6_3 + row_r7_3 + row_r8_3 + row_r9_3;
                        row3[j+1] =  row_i1_3 + row_i2_3 + row_i3_3 + row_i4_3 + row_i5_3 + row_i6_3 + row_i7_3 + row_i8_3 + row_i9_3;
                        row4[j]   =  row_r1_4 + row_r2_4 + row_r3_4 + row_r4_4 + row_r5_4 + row_r6_4 + row_r7_4 + row_r8_4 + row_r9_4;
                        row4[j+1] =  row_i1_4 + row_i2_4 + row_i3_4 + row_i4_4 + row_i5_4 + row_i6_4 + row_i7_4 + row_i8_4 + row_i9_4;
    }

        /*******  echo - FIDs stored in out1  ********/
        fwrite(row1, sizeof(int), td, fpout1);
        fwrite(row2, sizeof(int), td, fpout1);

        /*****  antiecho - FIDs stored in out2  ******/
        fwrite(row3, sizeof(int), td, fpout2);
        fwrite(row4, sizeof(int), td, fpout2);
}

/***** free resources *****/

free(row1);
fclose(fpin);
fclose(fpout1);
fclose(fpout2);

/***** store parameters  *****/

  td2  /= (split/2.0);
  td2s /= (split/2.0);

  byteorder = local_endian();

  DATASET(name, nexpno, procno, disk, user)
        STOREPARS("BYTORDA", byteorder)
    STOREPAR1("TD", td2)
    STOREPAR1S("TD", td2s)
    STOREPAR1S("FnMODE", 6)
    STOREPAR1("MC2", 5)
    STOREPAR3S("FnMODE", 6)
    STOREPAR3("MC2", 5)

  DATASET(name, nexpno+1, procno, disk, user)
        STOREPARS("BYTORDA", byteorder)
    STOREPAR1("TD", td2)
    STOREPAR1S("TD", td2s)
    STOREPAR1S("FnMODE", 6)
    STOREPAR1("MC2", 5)
    STOREPAR3S("FnMODE", 6)
```

```
      STOREPAR3("MC2", 5)

/****** re-sort echo and anti-echo FIDs into one dataset ******/

          DATASET (name,nexpno,procno,disk,user)

          GETCURDATA

          FETCHPARS("BYTORDA",&byteorder)
          FETCHPARS("TD", &tds)

          td = ( (tds + 255) / 256 ) * 256;

          FETCHPAR3("TD",&td1)
          FETCHPAR3S("TD",&td1s)

          FETCHPAR1("TD", &td2)
          FETCHPAR1S("TD", &td2s)

  (void)sprintf(infile10,"%s/data/%s/nmr/%s/%d/ser",disk,user,name,nexpno);
  (void)sprintf(infile20,"%s/data/%s/nmr/%s/%d/ser",disk,user,name,nexpno+1);

  (void)sprintf(outfile10,"%s/data/%s/nmr/%s/%d/ser",disk,user,name,newexpno);

          WRA(newexpno)

          fpin10=fopen(infile10,"rb");
          fpin20=fopen(infile20,"rb");
          fpout10=fopen(outfile10,"wb");

          for (j=0; j < td1s; j++)
          {
                    Show_status("recombining data");

          fread(row11,sizeof(int),td2s*td,fpin10);
          local_swap4(row11,sizeof(int)*td2s*td,byteorder);
          fread(row12,sizeof(int),td2s*td,fpin20);
          local_swap4(row12,sizeof(int)*td2s*td,byteorder);

          local_swap4(row11,sizeof(int)*td2s*td,byteorder);
          fwrite(row11,sizeof(int),td2s*td,fpout10);
          local_swap4(row12,sizeof(int)*td2s*td,byteorder);
          fwrite(row12,sizeof(int),td2s*td,fpout10);
          }

          Show_status("combining data finished");

          fclose(fpin10);
          fclose(fpin20);
          fclose(fpout10);

          td1 *= 2;
          td1s = (td1s * 2);

          DATASET (name,newexpno,procno,disk,user)
          STOREPAR3("TD",td1)
          STOREPAR3S("TD",td1s)

QUIT
```
